# Mental Dissolution

**Published:** 1900-02-17

**Authors:** G. H. Savage

**Affiliations:** at the Medical Graduates' College and Polyclinic on Wednesday, January 31st.


### Hospital Clinics and Medical Progress.

### MENTAL DISSOLUTION.

Abstract of a Clinical Lecture delivered by G. H. Savage, M.D., F.R.C.P., at the Medical Graduates' College and Polyclinic on Wednesday, January 31st.

Dr. Savage said the most powerful idea of the generation was evolution, and dissolution was sometimes considered as if it were merely the reversal of evolution, but that was only partially true. Much, however, could be learnt of evolution by the study of dissolution. He preferred as a definition of dissolution —separation into constituent parts or destruction of existing conditions. Dissolution then differed from evolution as being a passage to simpler states, whereas the latter was a passage to less definite states. It was necessary in the study of dissolution to include a temporary dissolution as well as the normal result of wear and tear; one saw the necessity of this in a similarity of the symptoms of alcoholic excess, and of the progressive brain decay of general paralysis. The degeneration in dissolution depended on the causes of destruction and on the stability of the structure, *i.e.*, minds built on the jerrybuilders' lines had little power of resistance, and were destroyed by any extra strain such as that of sexual development. The symptoms in this class were different to those of older patients. The class of alcoholic dissolution might be transient or permanent, and, if the latter, on the lines of the character of the transient condition. Restfulness was the perfect condition of the mind, and restlessness was one of the earliest signs of dissolution. This restlessness was most conspicuously seen in old ladies, who sometimes worried their households to distraction. Defective memory generally co-existed in such cases. This restlessness to him seemed to resemble an "itch" or skin irritability, and indeed such patients at times scratched themselves most indelicately without any regard to convention; there might be as well a local sexual organ irritability. Perpetual talking or logorrhœa was evidence of defective education, as seen in Dickens' characters, who said all they thought. The most cultured certainly considered that words, like Nature, should half reveal and half conceal the soul within. Restlessness and changing occupation was associated with "doubt" and inability to give a final decision. A rare and distressing symptom was that of swearing when addressed on any simple topic. Loss of temper, *i.e.*, loss of higher control, was often associated with the saying and doing things alien to one's nature, so with the dissolution of age or disease this might occur. Sensory hallucination was often present with dissolution, and this symptom was apt to give rise to trouble from legal or medico-legal points. Dr. Savage here illustrated his meaning by reference to two cases of visional hallucination. An intermediate state was sometimes present in cases of progressive dissolution—the hallucination affecting the patient generally on first awaking, or after an attack of a disease like influenza. Nervous breakdown and sexual perversions were sometimes preceded by marked hallucination of smell. Loosening of the mental unity was one of the most characteristic signs of mental dissolution. The individual had been fitted into a certain groove more or less specialised; with the advance of dissolution he became a bad fit, and ceased to be himself as before. Self-neglect was very commonly met with in these cases, as were also laziness and dirtiness. Neglect of morality was probably the most troublesome symptom. Men of pure lives consorted with designing women and became their prey. A patient of his thought it not strange to travel openly with his (the patient's) mistress, and at the same time wrote daily to his (patient's) wife in affectionate terms, and in the letters openly describing what *they* were doing. It was therefore to be remembered that persons in the earlier stages of mental dissolution became changed in every way, so that they passed from the moral to the immoral. Sexual desire became uncontrolled; he was often consulted about persons so failing doing indecent acts. In such cases the judge and jury might punish most cruelly one who was irresponsible.

Memory was commonly regarded as the gauge of dissolution, but such defect might occur quite independently of any general process of mental decay, and, in cases of progressive loss of mental power, memory was sometimes retained. The memory seemed to be driven back in separate stages till only events of early childhood could be recalled. The decay spread from the isolated independent memory to that which was more organised and associated. There appeared to be an increase in the accuracy of the memory of the long past. He had been able to show the probability of a clearing up of memory by denudation. Some patients had gaps in their memories, and these confused the past with the present; these cases almost might give rise to serious legal complications. Defective memory did not necessarily accompany progressive brain diseases. This was shown in general paralysis of the insane, where the patient's memory was sometimes remarkably good. Again, almost absolute defect of recent memory might be practically the only symptom of mental weakness, as was often seen in alcoholics. Then there were particular forms of memory, and special defects which might occur in them in old age; he had, for instance, met persons who had no memory as to their appetite. An apparent increase in vigour was not infrequently associated with dissolution, which was hard to explain. The active-minded man did not show signs of decay earlier than the more placid. There were few people in whom dissolution was started by overwork, but many in whom it was started by retiring from active work. Patients often develop hypochondriasis and melancholia amid the other signs of evolution, pointing to the decay felt by the patient. Probably the fear of the workhouse or of ruin was the generally-accepted interpretation of the morbid feeling of dissolution. It occurred chiefly in men and women who had to work hard for their living. Then there were the fretful, suicidal people, with whom reason and evidence were of no avail; it might result in melancholic ruin. Certain families, whose members are long-lived, nearly always ended life with prolonged depression. No sign of decay occurred in these till they were between 70 and 80, and finally melancholia resulted. One had to remember that loss of control of he emotions was more prevalent with the old, but the feeling was not increased, only the expression. Another exhibition of loss of control was in the violent passions arising from small causes in many old persons; it was necessary to divert the individual's attention like one would do with a child. Dissolution, then, might be regular in its progress, or irregular in its rate or in the parts affected, so that one could not use any one faculty as a gauge of the depth of the dissolution; the effects would vary with the patient's age and hereditary disposition. Finally, he would wish to impress upon his hearers that dissolution was not a hopeless condition, and that many with marked defects might, by fresh methods of accommodation, carry on life's battle to the end.

